# Mechanistic convergence and shared therapeutic targets in Niemann‐Pick disease

**DOI:** 10.1002/jimd.12191

**Published:** 2019-12-05

**Authors:** Alexandria Colaco, Ecem Kaya, Elias Adriaenssens, Lianne C. Davis, Stefania Zampieri, María E. Fernández‐Suárez, Chong Y. Tan, Patrick B. Deegan, Forbes D. Porter, Antony Galione, Bruno Bembi, Andrea Dardis, Frances M. Platt

**Affiliations:** ^1^ Department of Pharmacology University of Oxford Oxford UK; ^2^ University Hospital Santa Maria della Misericordia Udine Italy; ^3^ Lysosomal Disorders Unit Addenbrooke's Hospital Cambridge UK; ^4^ *Eunice Kennedy Shriver* National Institute of Child Health and Human Development, NIH Bethesda Maryland

**Keywords:** ABCA1, lysosome, Niemann‐Pick disease type C, substrate reduction therapy, Tangier disease

## Abstract

Niemann‐Pick disease type C (NPC) and Tangier disease are genetically and clinically distinct rare inborn errors of metabolism. NPC is caused by defects in either *NPC1* or *NPC2*; whereas Tangier disease is caused by a defect in *ABCA1*. Tangier disease is currently without therapy, whereas NPC can be treated with miglustat, a small molecule inhibitor of glycosphingolipid biosynthesis that slows the neurological course of the disease. When a Tangier disease patient was misdiagnosed with NPC and treated with miglustat, her symptoms improved. This prompted us to consider whether there is mechanistic convergence between these two apparently unrelated rare inherited metabolic diseases. In this study, we found that when *ABCA1* is defective (Tangier disease) there is secondary inhibition of the NPC disease pathway, linking these two diseases at the level of cellular pathophysiology. In addition, this study further supports the hypothesis that miglustat, as well as other substrate reduction therapies, may be potential therapeutic agents for treating Tangier disease as fibroblasts from multiple Tangier patients were corrected by miglustat treatment.

## 1 INTRODUCTION

Niemann‐Pick disease type C (NPC) is a rare inborn error of metabolism caused by mutations in either *NPC1* or *NPC2*. Cellular hallmarks of NPC include sphingolipid storage and mis‐trafficking, an expanded late endosomal/lysosomal compartment and reduced levels of lysosomal Ca^2+^ leading to impaired Ca^2+^ signalling that effects late endosome/lysosome fusion.^1^ The relationship between NPC1 and NPC2 remains incompletely understood. However, these two proteins are thought to be involved in a cholesterol transport pathway from lysosomes or to be part of a cholesterol‐regulated pathway that promotes lipid/substrate movement from the lysosome.^2^, ^3^

Tangier disease (familial alpha‐lipoprotein deficiency) is an ultra‐rare inborn error of metabolism resulting in defective cholesterol efflux from cells and is caused by mutations in the ATP‐binding cassette transporter protein A1 (ABCA1).^4^ ABCA1 regulates cellular cholesterol and phospholipid homeostasis by transferring lipids across the plasma membrane to extracellular acceptors.^5^ Typically, ABCA1 transfers cholesterol to apolipoproteins, particularly apoA‐1, to form high‐density lipoproteins (HDL) particles.^6^ As a consequence, Tangier disease patients have decreased serum levels of HDL and cholesterol.^7^ Diagnosis is often based on characteristically enlarged, orange coloured tonsils and peripheral neuropathy.^7^ However, due to the small number of patients described to date the full spectrum of clinical manifestations in this disease remains incompletely defined, as does its true incidence.

Multiple studies have linked changes in ABCA1 expression/function to changes in the expression of *NPC1* and *NPC2*.^8^ As ABCA1 and the NPC1/NPC2 proteins are involved in regulating different aspects of cellular cholesterol homeostasis, this is not unexpected as it likely reflects perturbations and compensatory responses to changes in cholesterol homeostasis within diseased cells.^9^ For example, increased expression of ABCA1 in NPC1 deficient cells using upstream activators, such as LXR, increases cholesterol and lipid efflux thus rescuing the cellular phenotypes characteristic of NPC disease cells.^10^ The majority of NPC patients have low plasma HDL‐cholesterol and NPC is the only disease identified to date that has low HDL levels as a consequence of reduced ABCA1 protein expression, rather than a mutation in ABCA1 as seen in Tangier disease.^8^ The impaired HDL levels may be due to the accumulation of glycosphingolipids (GSLs) that occur in this disease, as GSL storage has previously been shown to inhibit apoA‐1‐mediated cholesterol efflux.^11^ It has also been suggested that the NPC1 pathway may play a role in vesicular transport of drugs and may have overlapping function with ABC transporters.^12^

However, a case report highlighted for the first time that there may be a mechanistic link between cellular pathogenesis in NPC and Tangier disease. An adult patient presenting with an atypical late‐onset form of Tangier disease was misdiagnosed with NPC and put on the current EMA approved treatment, miglustat.^13^ After 4 months of treatment the patient's neurological and dermatological symptoms significantly improved, and was subsequently correctly diagnosed with Tangier disease after genetic testing.^13^

The question this case report poses is if there is there a stronger mechanistic connection between ABCA1 and NPC1 that underlies the response of the Tangier patient to an NPC therapy. We have therefore investigated whether a failure in ABCA1 function leads to secondary inhibition of the NPC disease pathway. If this were the case, we would predict that the cellular hallmarks of NPC disease would be present in Tangier disease patient cells, in addition to the primary Tangier disease defects.

## 2 RESULTS

**Figure 1:**
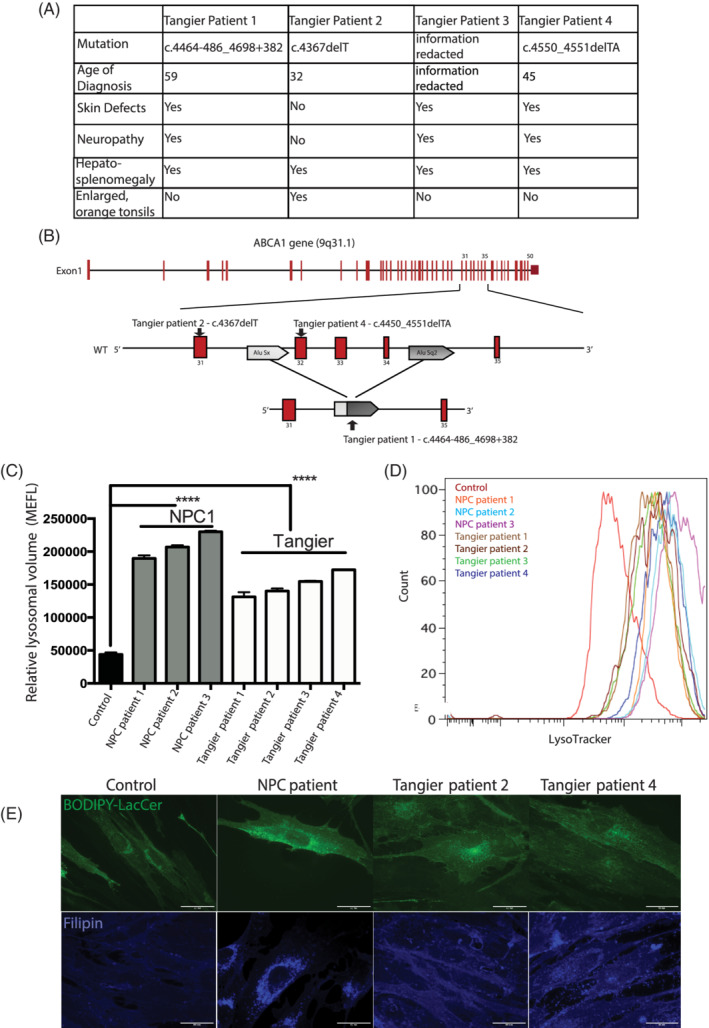
Tangier patient fibroblasts have enlarged lysosomal compartment, and cellular phenotypes consistent with lysosomal storage disorders. A, Summaries of Tangier patient details, including mutations, age of diagnosis, and phenotypes. B, ABCA1 gene schematic indicating mutation placement. C, Relative lysosomal volume by LysoTracker staining showed significant increase in volume in the four Tangier patient cells with (D) representative traces. E, BODIPY‐LacCer trafficking and cholesterol accumulation were observed in Tangier cells 1 and 2 and resembled the phenotype observed in NPC1 patient cells

We first investigated whether fibroblasts from the Tangier disease patient who responded to miglustat therapy^13^ and three additional Tangier disease patients shared biochemical and cellular phenotypes observed in NPC1 patient fibroblasts. These patients do have some heterogeneity with regards to their clinical features (Tangier patient clinical details in Figure 1A), however the mutations all lead to a shift in the open reading frame and the generation of a premature stop codon resulting in a truncated ABCA1 protein (Figure 1B).

Although many NPC cellular phenotypes are not unique to this disease, when viewed in combination they define NPC disease at the cellular level and those measured in this study include lysosomal compartment size, sphingolipid trafficking, lipid storage, and acidic store calcium levels. We investigated whether some or all of these cellular phenotypes were present in the Tangier disease patient fibroblasts.

### 2.1 Enlarged acidic compartment in Tangier disease fibroblasts

The four Tangier disease patient‐derived fibroblasts were examined to see if the acidic compartment was enlarged, as is observed in NPC.^14^ We measured an increase in relative lysosomal volume using flow cytometry (approximately threefold increase; control vs Tangier patient 1 *P* < .0001, control vs Tangier patient 2 *P* < .0001, control vs Tangier patient 3 *P* < .0001, control vs Tangier patient 4 *P* < .0001) similar to that observed in NPC1 disease cells (approximately fivefold increase; control vs NPC1 patient 1 *P* < .0001; control vs NPC1 patient 2 *P* < 0.0001; control vs NPC1 patient 3 *P* < .0001; Figure 1C, representative FACs trace Figure 1D).

### 2.2 Sphingolipid mis‐trafficking and GSLs, cholesterol and free fatty acids storage in Tangier disease fibroblasts

As NPC disease is a lysosomal storage disorder characterised by lipid storage in LE/Lys, sphingolipid mis‐trafficking and impaired acidic store Ca^2+^ levels^1^, ^15^ we studied whether these parameters were also present in Tangier disease patient fibroblasts.

We investigated sphingolipid trafficking in Tangier disease patient fibroblasts using a BODIPY‐LacCer trafficking assay and observed defective endocytic trafficking of exogenously administered BODIPY‐LacCer as previously described in NPC and other lysosomal storage diseases.^16^, ^17^ Golgi staining was observed in control fibroblasts (Figure 1E) consistent with sphingolipid recycling, whereas NPC1 and Tangier disease fibroblasts exhibited a punctate distribution that has previously been shown to be LE/Lys (Figure 1E).^16^, ^18^ Additionally, filipin (cholesterol) staining indicated intracellular cholesterol storage (Figure 1E) in the Tangier patient fibroblasts. However, these cells showed a milder cholesterol accumulation when compared with the classical biochemical NPC1 phenotype, displaying a pattern similar to that observed in NPC fibroblasts with the so‐called variant phenotype.

**Figure 2:**
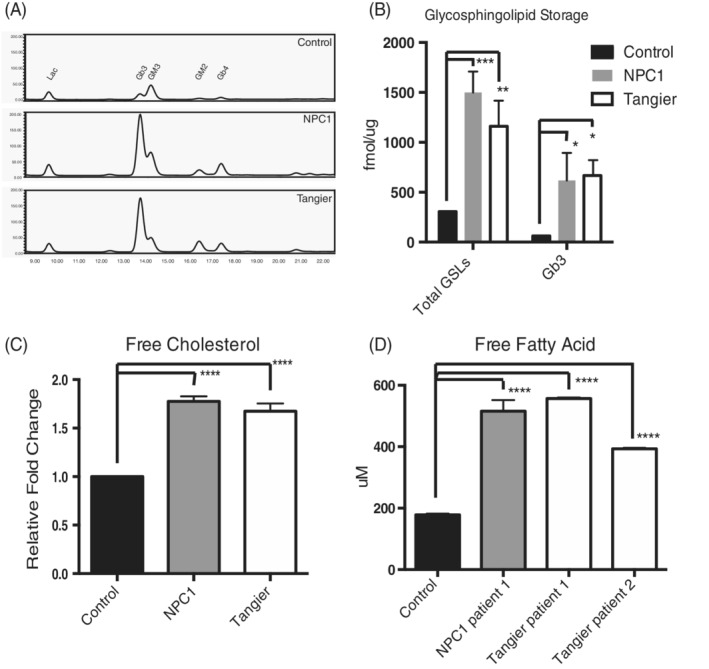
Tangier patient fibroblasts accumulate glycosphingolipids, cholesterol and free fatty acids. A, Representative glycosphingolipid (GSLs) traces from control, NPC1, and Tangier patient fibroblasts lines and B, the mean ± SEM for total GSLs and main storage species Gb3; 3 control, 3 NPC1, 4 Tangier, ***P < .001 vs control, **P < .01 vs control, *P < .05 vs control, calculated by a two‐way ANOVA test. C, Free cholesterol was measure from the patient cells and data presented as the mean ± SEM, ****P < .0001 vs control. D, Free fatty acids were measure from individual patient cell lines and presented as the mean, n = 3, ****P < .0001 vs control

Biochemical analysis was performed to quantify lipid storage. An accumulation of GSLs, free fatty acids^19^ and cholesterol was observed in all four Tangier disease fibroblasts. Total GSLs were elevated approximately fourfold in Tangier and nearly fivefold in NPC1 patient cells (representative HPLC traces in Figure 2A,B). Levels of globotriaosylceramide (Gb3), which is the most abundant GSL species in human fibroblasts, were significantly elevated (Figure 2B; Total GSLs: control vs Tangier *P* = .0019, control vs NPC1 *P* = .0001; Gb3: control vs Tangier *P* = .0214, control vs NPC1 *P* = .0408).

Similarly, a significant increase in cholesterol (>1.5‐fold elevation in both NPC and Tangier fibroblasts, Figure 2C; Free cholesterol: control vs Tangier patient 1 *P* < .0001, control vs NPC1 patient 1 *P* < .0001, control vs NPC1 patient 2 *P* < .0001) and free fatty acids (2‐3‐fold increase in both NPC1 and Tangier, Figure 2D; Free fatty acids: control vs Tangier patient 1 *P* < .0001, control vs Tangier patient 2 *P* < .0001, control vs NPC1 patient 1 *P* < .0001) were observed.

### 2.3 Lysosomal calcium defect present in Tangier disease fibroblasts

**Figure 3:**
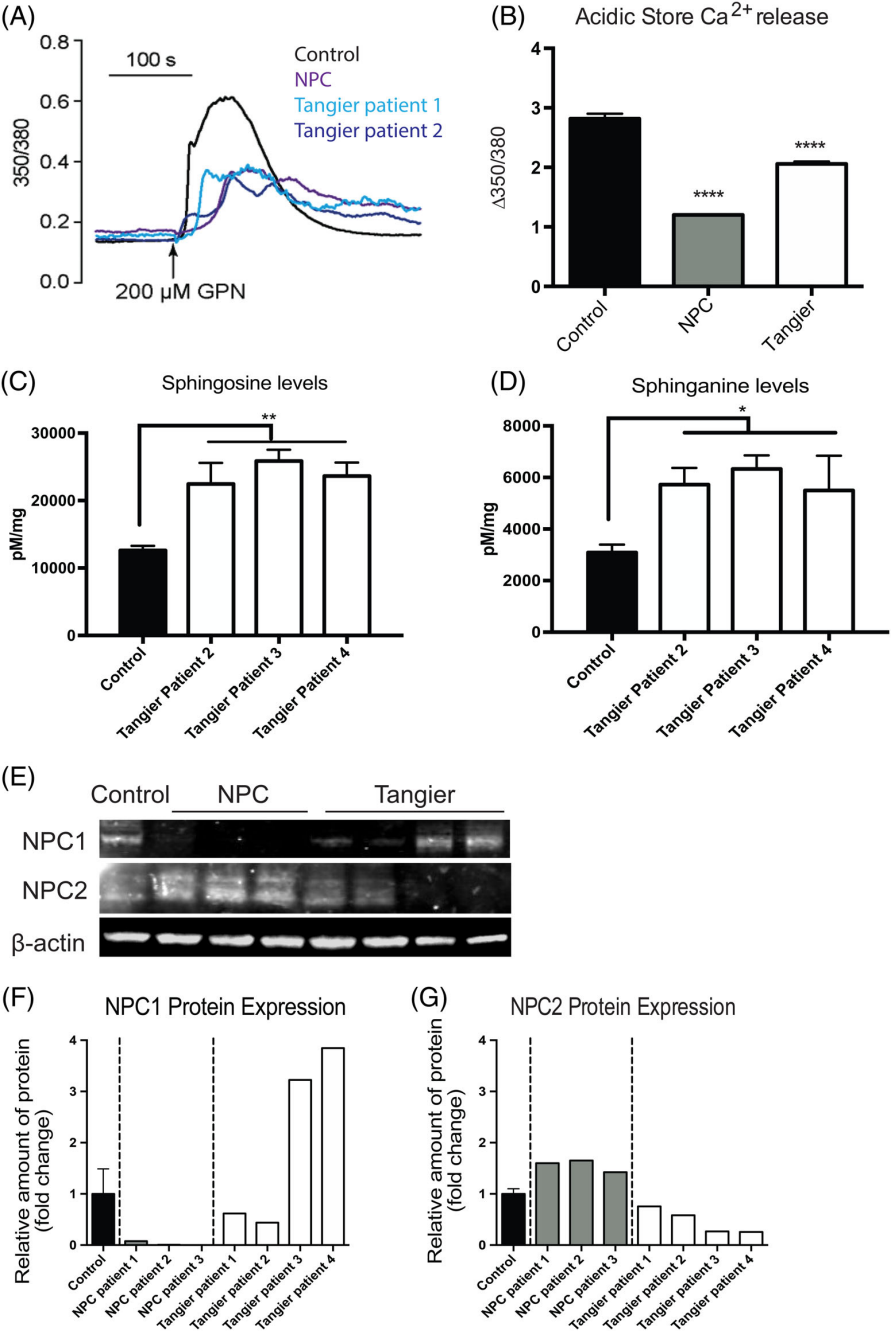
Tangier patient fibroblasts have reduced acidic store calcium and significant sphingosine and sphinganine storage, and altered NPC1 and NPC2 expression. A, Representative traces showing intracellular [Ca^2+^] changes monitored in single fura‐2‐loaded fibroblasts in response to 200 μM GPN. B, Maximal peak fluorescence changes were determined as the difference between basal and the maximum fluorescence (**Δ**350/380). Data are presented as the mean ± SEM; 66 control, 81 NPC1, 78 Tangier patient 1, and 72 Tangier patient 2 cells, *** *P* < .001 vscontrol, calculated by an one‐way ANOVA test. C, Sphingosine (C18:1) and D, Sphinganine (C18:0) levels were measured in patient fibroblasts by HPLC. Date presented as mean ± SEM, n = 3, *P < .05, **P < .01, calculated by a one‐way ANOVA test. E, Western blot analysis of three NPC1 patient and four Tangier patient fibroblast lines. The NPC1 patients all had little/no level of NPC1, and normal/high levels of NPC2. The Tangier patients had variable levels of the proteins, with patients 1 and 2 having low levels of NPC1 and patients 3 and 4 having a high expression level. Similarly, Tangier patients 1 and 2 had normal/low levels of NPC2 whereas tangier patient 3 and 4 had reduced levels of NPC2 expression. (F) Data presented as a fold‐change of the relative amount of protein to the control for NPC1 protein levels and G, NPC2

NPC1 is characterised in part by reduced acidic store Ca^2+^ levels, which was previously measured using calcium release assays and calcium sensors within LE/Lys.^1^ As the Tangier patient derived fibroblasts shared many of the downstream defects and lipid storage phenotypes observed in NPC, we also measured acidic store Ca^2+^ release in response to GPN (glycyl‐l‐phenylalanine 2‐naphthylamide) in Tangier disease cells. Of the two patient cell lines examined, both patients exhibited significantly reduced acidic store Ca^2+^ release in response to GPN as compared to control cells (Figure 3A,B; 30% to 40% reduction in Tangier patients, 50% to 60% reduction in NPC1 patients; control vs Tangier disease patients *P* < .0001, control vs NPC patients *P* < .0001). In the case of NPC1, this reduction in acidic store Ca^2+^ has been attributed to the accumulation of lysosomal sphingosine.^1^

### 2.4 Elevated levels of sphingosine in Tangier disease fibroblasts

Sphingosine storage has been shown to lead to the drop in lysosomal Ca^2+^ levels in NPC cells.^1^, ^15^ We therefore investigated if sphingosine levels are also elevated in Tangier disease cells and found them to be significantly higher in the Tangier patient fibroblasts (Figure 3C; control vs Tangier patient 2 *P* = .0027; control vs Tangier patient 3 *P* = .0002; control vs Tangier patient 4 *P* = .0008). We also examined if sphinganine levels were elevated in the patient fibroblasts, as this had also been previously shown to be stored in both liver and spleen from NPC patients.^15^ We found that sphinganine levels were significantly higher in Tangier patient fibroblasts relative to controls (Figure 3D; control vs Tangier patient 2 *P* = .0167; control vs Tangier patient 3 *P* = .0051; control vs Tangier patient 4 *P* = .0264).

### 2.5 Relationship between NPC1, NPC2, and ABCA1

When we measured levels of NPC1 and NPC2 in Tangier disease cells by western blotting we found there to be variation between the patients (Figure 3E). Tangier patients 3 and 4 both had significant up‐regulation of NPC1 and a trend towards down‐regulation of NPC2, in agreement with previous findings^20^ suggesting that Tangier cells express higher levels of NPC1 relative to controls (Figure 3F,G). However, Tangier patient 1 and 2 fibroblasts did not have altered NPC1 or NPC2 protein expression (Figure 3F,G), despite having reduced acidic store Ca^2+^ and sphingosine, GSL, cholesterol and fatty acid accumulation.

### 2.6 Efficacy of substrate reduction treatment in Tangier disease

As the initially misdiagnosed Tangier patient improved clinically following miglustat treatment,^13^ we studied the effects of miglustat at the cellular and biochemical level in Tangier disease cells from all four patients.

**Figure 4:**
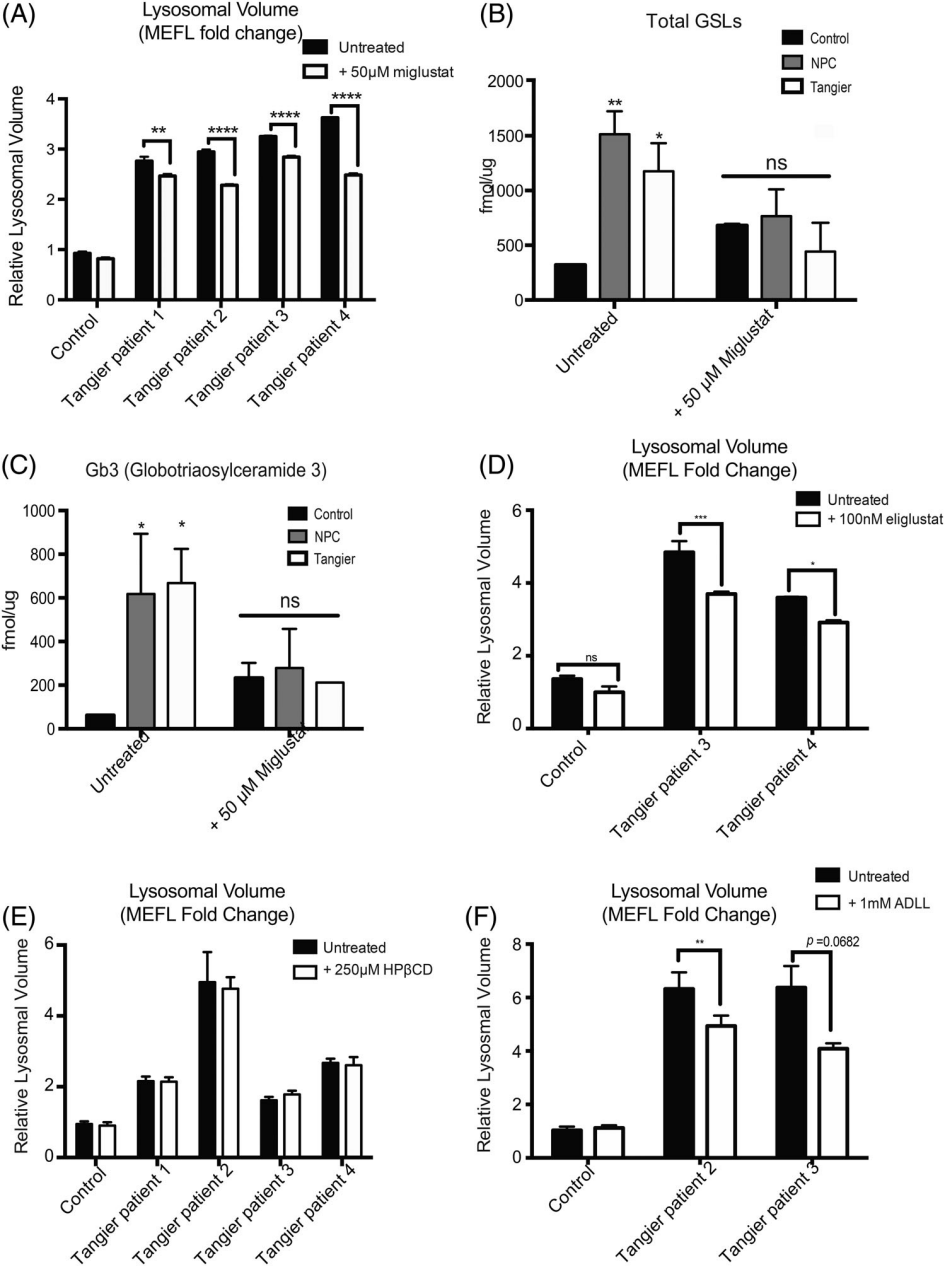
Tangier patient cells respond to substrate reduction therapy (miglustat and eliglustat) treatment, acetyl‐d‐leucine (ADLL) treatment but not HPβCD. A, Relative lysosomal volume by LysoTracker staining showed significant reduction in volume in the four Tangier cell lines following 50 μM miglustat treatment. B, Total levels of glycosphingolipids following miglustat treatment were not significantly elevated from control levels. C, Similar to the levels of Gb3, which following miglustat treatment were not significantly different in the NPC1 and Tangier fibroblasts. D, Significant reduction in relative lysosomal volume in Tangier patient 3 and 4 cell lines was observed following 100 nM eliglustat treatment. E, 250 μM HP*β*CD treatment did not have any significant effect on relative lysosomal volume in any of the four Tangier patient cells. F, Treatment with 1 mM ADLL significantly reduced the relative lysosomal volume by LysoTracker staining in Tangier patient 2 and 3 cells

Following 50 μM miglustat treatment for 72 hours we observed a significant reduction in relative lysosomal volume measured by flow cytometry using LysoTracker staining. (Figure 4A; Tangier patient 1 UT vs Tangier patient 1 + 50 μM miglustat *P* = .0015; Tangier patient 2 UT vs Tangier patient 2 + 50 μM miglustat *P* < .0001; Tangier patient 3 UT vs Tangier patient 3 + 50 μM miglustat *P* < .0001; Tangier patient 4 UT vs Tangier patient 4 + 50 μM miglustat *P* < .0001). As LysoTracker staining has been shown to be a biomarker for NPC1,^14^ these data suggest that miglustat treatment is having a therapeutic effect on the Tangier patient fibroblasts.

Following miglustat treatment total GSL levels in all Tangier patient fibroblasts returned to control levels, as did Gb3, the most highly expressed GSL species in human fibroblasts (Figure 4B; Total GSLs: control +50 μM miglustat vs Tangier patients +50 μM miglustat *P* = 0.4336; Figure 4C Gb3: control +50 μM miglustat vs Tangier patients +50 μM miglustat *P* = 0.9891).

Eliglustat tartrate and miglustat are both substrate reduction therapy drugs that act by inhibiting GSL biosynthesis, but differ in their off‐target effects.^21^ As Tangier disease patients do not have CNS pathology, the inability for eliglustat to distribute into the brain should not compromise the drug's efficacy in Tangier patients.^22^ Following 6 days of incubation with 100 nM eligustat, we observed a significant reduction in lysosomal volume measured by LysoTracker staining (Figure 4D; Tangier patient 3 UT vs Tangier patient 3 + 100 nM eliglustat *P* < .0001; Tangier patient 4 UT vs Tangier patient 4 + 100 nM eliglustat *P* = .0009).

### 2.7 Efficacy of other NPC1 investigational therapies, HPβCD, and acetyl‐DL‐leucine (ADLL), in Tangier disease

We also investigated whether other experimental NPC therapies would have similar therapeutic efficacy in Tangier disease cells as it may provide insights into the underlying pathogenic/convergent mechanisms.^9^ We therefore examined the effects of 2‐hydroxypropyl‐β‐cyclodextrin (HPβCD), which reduces cholesterol and sphingolipid storage and is currently in clinical trials for NPC1,^23^ as well as acetyl‐DL‐leucine (ADLL), which has previously been shown to improve symptoms in patients with cerebellar ataxia.^24^

The effects of HPβCD are not fully understood although it has been shown to enhance exocytosis.^25^, ^26^ We observed no changes in lysosomal volume measured by LysoTracker staining following HPβCD treatment for 24 hours (Figure 4E; Tangier patient 1 UT vs Tangier patient 1 + 250 μM HPβCD *P* > .9999; Tangier patient 2 UT vs Tangier patient 2 + 250 μM HPβCD *P* = .9652; Tangier patient 3 UT vs Tangier patient 3 + 250 μM HPβCD *P* = .9776; Tangier patient 4 UT vs Tangier patient 4 + 250 μM HPβCD *P* = .9998).

ADLL is a well‐tolerated drug, and in an observational study was shown to improved the ataxic symptoms in 12 NPC1 patients, however the mechanism of action in NPC has not yet been determined.^27^ Following 6 days of incubation with 1 mM ADLL, we observed a significant reduction in lysosomal volume as measured by LysoTracker staining in patient fibroblasts (Figure 4F; Tangier patient 2 UT vs Tangier patient 2 + 1 mM ADLL *P* = 0.0074; Tangier patient 3 UT vs Tangier patient 3 + 1 mM ADLL *P* = 0.0682;).

## 3 DISCUSSION

In this study, we have found that Tangier disease shares several cellular characteristics with those of the lysosomal storage disorder NPC1. Abnormalities in the lysosome have been previously observed in Tangier disease cells, including lysosomal accumulation of cholesterol and sphingomyelin.^20^, ^28^ It may be a disorder of intracellular trafficking as HDL receptors are not recycled but are stored in the lysosomal compartment.^29^ It has been suggested that ABCA1 plays a role in cellular lipid efflux in the late endocytic system, specifically by mediating apoA‐1 lipidation and trafficking of lipids to the cell surface from the late endocytic system.^20^ Additionally, there have also been previous investigations reporting links between NPC and Tangier disease, with a focus on the potential interplay between NPC1 and ABCA1 where it was found that ABCA1 can mobilise cholesterol and bypass the NPC1 mutation in NPC disease cells.^30^

Strengthening these previous findings, in this study we have found that all of the cellular features of NPC are recapitulated in Tangier disease patient cells, suggesting a reclassification of Tangier disease as a secondary lysosomal storage disorder. While Tangier disease cells share some traits with all lysosomal disorders such as enlarged lysosomes, we also found endocytic mis‐trafficking of sphingolipids; storage of cholesterol, sphingosine, GSLs, free fatty acids and sphingomyelin, as well as the acidic store Ca^2+^ defect characteristic of NPC. The combination of the high levels of sphingosine storage and reduced lysosomal calcium levels are features that are unique to NPC relative to other sphingolipidoses. The pathogenic cascade in NPC1 has been previously been shown to be triggered by the storage of sphingosine,^1^ suggesting that it is likely that the cellular pathology we observe in Tangier disease is also due to sphingosine storage. Interestingly, ABCA1 has been implicated to act as a sphingosine‐1‐phosphate exporter,^31^, ^32^ and studies have suggested that cholesterol efflux via ABCA1 is regulated in part by sphingosine kinase.^33^ Although the mechanism leading to elevated levels of sphingosine in Tangier disease remains unknown, it is plausible that sphingosine storage is a direct consequence of mutations in ABCA1 considering the interrelationship between ABCA1, sphingosine kinase, and spingosine‐1‐phosphate.^32^, ^33^

In this study, we observed complete rescue of phenotypes observed in Tangier disease following 50 μM miglustat treatment. Why reducing GSL levels with miglustat or inhibiting GBA2, an additional target of miglustat,^34^ in NPC is therapeutically beneficial is still poorly understood in NPC, and is equally enigmatic in Tangier disease. GSL biosynthesis inhibitor therapies (miglustat, eliglustat, and so on), may be particularly effective as it has been previously demonstrated that GSL accumulation can further inhibit ABCA1 expression, and in this way may have increased efficacy in Tangier disease patients.^11^

An interesting difference between NPC1 and Tangier was the lack of efficacy of HPβCD in Tangier patient fibroblasts as it is very effective in the treatment of NPC disease animal models and corrects NPC cells.^35^ Previous studies reported that cholesterol storage could not be depleted in Tangier cells following HPβCD treatments,^20^ in line with our findings. Furthermore, a recent study found the mRNA expression of ABCA1 to be significantly increased in *Npc1* ^−/−^ liver treated with HPβCD,^36^ suggesting that ABCA1 may be a direct target of HPβCD. Our findings will be useful in further elucidating through which mechanisms HPβCD is effective in the treatment of NPC disease, as these data suggest it may function through an ABCA1‐dependent mechanism.

The convergent cellular pathology between these apparently unrelated human metabolic disorders has important implications for our understanding of the consequences of dysregulated lipid homeostasis and for developing therapies to treat these rare diseases.

By better understanding the role of ABCA1 in NPC disease, we can potentially identify drugs that upregulate ABCA1 to increase lipid efflux and alleviate the lysosomal burden. Additionally, through a better understanding of the functions of ABCA1 in the endocytic system^37^ and at the plasma membrane, we will gain insights into Tangier disease, as well as NPC. As the fibroblasts from the patient in the initial case report and all three additional patient fibroblasts evaluated in this study were responsive to the GSL biosynthesis inhibitors miglustat and eliglustat, these substrate reduction therapy drugs may be unanticipated potential disease modifying therapies for the previously untreatable Tangier disease.

## 4 MATERIALS AND METHODS

### 4.1 Patients derived fibroblasts

Human ABCA1‐mutant fibroblast cultures, surplus to the requirements for diagnosis, were received from the University Hospital‐Udine and Addenbrooke's Hospital, Cambridge and anonymized. Patients 1, 2, 4 all gave written informed consent for the publication of their details in this case report. Patient 3 was lost to follow up and so all potentially identifiable personal data has been redacted. Patient details are summarised in Figure 1A. Mutations were found in homozygosity in the four Tangier disease patient‐derived fibroblasts (Tangier patient 1: ABCA1 c.4464‐486_4698+382, Tangier patient 2: ABCA1 c.4367delT, Tangier patient 3 [information redacted], Tangier patient 4: ABCA1 c.4450_4551delTA) all lead to a shift in the open reading frame and the generation of a premature stop codon resulting in a truncated ABCA1 protein. NPC1 patient cells were obtained from Dr Porter at the NIH and patient cell line mutations were: c.3182 T>C, 3556C>G (p.I1061T, R1186G); c3176G>A, c3742_3745delCTCA (p.R1059Q fs exon24); c.2979dupA| C2103C>G (p.N701K fs exon 20). The fibroblasts were maintained in DMEM with 10%FCS, 1% penicillin/streptomycin and 1% l‐glutamine. All cells were cultured at 37°C with 5% CO_2_. Cell treatment with miglustat (*N*B‐DNJ) 50 μM for 72 hours, eliglustat 100 nM 6 days, HPβCD (H107, Sigma Aldrich) 250 μM 24 hours, ADLL 1 mM 6 days.

### 4.2 Flow cytometry

Relative lysosomal volumes was measured from patient derived fibroblasts as previously described.^14^ Live cells were washed twice with PBS and stained in triplicate using LysoTracker green (200 nM PBS) for 10 minutes at 20°C. Cells were moved into FACs buffer (100 μL 10% BSA, 100 μL 2 M NaN3 per 10 mL PBS) and PI stained (1 μg/mL) immediately prior to FACS analysis. FACS analysis was performed on a BD FACSCantoII flow cytometer with BD Bioscience FACSDiva software, with 10.000 cell events recorded. The molecules of equivalent fluorescence (MEFL) were calculated using 8‐peak Rainbow calibration beads (BD), using the fluorescein equivalent values provided.

### 4.3 BODIPY‐lactosylceramide transport/Cholera toxin subunit B transport

We performed BODIPY‐lactosylceramide transport assays as previously described^38^ with minor modifications. We used BODIPY‐LacCer (Molecular Probes) at a concentration of 5 μM with an initial incubation for 30 minutes at 20°C followed by a 2‐hour chase at 37°C and three 5‐minutes washes in medium containing 10% FCS and 1% BSA. Cholera Toxin subunit B (CTxB, binds GM1 ganglioside) transport was performed as previously described^39^ with minor modifications. Cells were incubated with 1 μg/mL CTxB for 30 minutes at RT, washed, and incubated for 90 minutes with fresh media, 1% BSA at 37°C. After incubation cells were washed 3× fresh media, fixed with 4% paraformaldehyde and visualised.

### 4.4 GSL analysis

HPLC measurements were performed as previously described^40^ with minor modifications. Briefly, GSLs were extracted from cell homogenates (1 mg protein) in C:M 1:2 overnight. The mixture was centrifuged and two parts chloroform and two parts PBS were added to the supernatant and centrifuged. The lower phase was dried and resuspended in C:M 1:3 and mixed with the upper phase. GSLs were recovered using C_18_ Isolute columns (100 mg, Biotage), and the column elutant was dried and resuspended in ceramide glycanase buffer (50 mM sodium acetate pH 5.5, 1 mg/mL sodium taurodeoxycholate). CGase (50 mU, Orphazyme APS) was added and sample incubated overnight. Released lipids were anthranilic acid (2‐AA) labelled and purified on Discover DPA‐6S columns (SUPELCO). Lipids were eluted in H_2_O and loaded 60:140 H_2_O: MeCN (v/v) for normal phase high‐performance liquid chromatography (HPLC).

### 4.5 Sphingosine analysis by HPLC

Sphingoid bases were extracted from homogenised cells (10 mg w/w) in 100uL H_2_O and spiked with an internal standard [C20, 3uL 0.1 mM]. To the homogenate, 500 μL C:M 1:2 was added and sonicated for 10 minutes, RT. To the samples 500uL 1 N NaCl, 500 μL chloroform, and 100uL 3 M NaOH were added and incubated for 15 minutes, RT and vortexed every 5 minutes. Samples were then centrifuged (13 000 *g*/10 minutes) and the lower phase purified on SPE NH2 columns [Biotage] pre‐equilibriated with 2 × 1 mL chloroform and eluted with 3 × 300 μL acetone. The column elutant was then dried down under N_2_, and resuspended in 50 μL pre‐warmed HPLC grate EtOH. Sphingoid bases were labelled with 50 μL OPA labelling solution (o‐phtalaldyhyde dissolved in 20x EtOH, 1× 2‐mercaptoethanol and diluted in 2000× 3% boric acid pH 10.5) and incubated at 20**°**C for 20 minutes, vortexing at 10 minutes intervals. Samples were buffered in 100 μL MeOH:5 mM Tris pH 7 9:1, centrifuged (5000*g*/2 minutes), and the supernatant was loaded for normal phase HPLC. Solvent A was MeOH, solvent B was H_2_O, solvent C was MeCN, and solvent D was MeCN:H_2_O 1:4. Separation was carried out using Hitachi L‐2485 FL Detector, excitation 340, and emission 455.

### 4.6 Intracellular Ca^2+^ measurements

Cells were loaded with 2 μM Fura‐2/AM in the presence of 0.03% Pluronic F127 in extracellular medium (ECM, mM: 121 NaCl, 5.4 KCl, 0.8 MgCl_2_, 1.8 CaCl_2_, 6 NaHCO_3_, 25 HEPES, 10 Glucose) for 45 minutes at 20°C followed by a 15 minutes de‐esterification. Cells were imaged in ECM using an Olympus IX71 microscope equipped with a ×40 UApo/340 objective and a 12‐bit Photometrics Coolsnap HQ2 CCD camera. Cells were excited alternately by 350‐ and 380‐nm light using a Cairn monochromator; emission was collected at 480 to 540 nm. The lysosomal Ca^2+^ content was assessed upon addition of 200 μM GPN. Autofluorescence was determined at the end of each run by addition of 1 μM ionomycin with 4 mM MnCl_2_ to quench fura‐2. Experiments were conducted at room temperature with an image collected every 2 to 3 seconds. Images were analysed using custom‐written Magipix software (Ron Jacob, King's College London, UK) on a single‐cell basis, the autofluorescence was subtracted and the data expressed as the mean ± SEM of the maximum peak fluorescence changes (Δ350/380).

### 4.7 Cholesterol measurements

Cholesterol and cholesterol esters were quantified using Amplex Red (Molecular Probes) according to the manufacturer's instructions. We visualise cellular cholesterol with filipin (Polysciences) as previously described.^38^

### 4.8 NPC1 and NPC2 protein levels

Protein levels were assessed by western blots using 30ug of protein. NPC2 primary antibody (sc‐30 346, Santa Cruz Biotechnology) was used at a dilution of 1:200, and NPC1 primary antibody (sc‐20 152, Santa Cruz Biotechnology) was used 1:1000.

### 4.9 Statistical analysis

The data were analysed using one‐way/two‐way ANOVA with Tukey's post‐hoc multiple comparison test to compare all sets of data as appropriate. Statistical analysis was performed with GraphPad Prism.

## CONFLICT OF INTEREST

F.M.P. consults for Actelion, F.M.P. and A.G. are co‐founders and consultants to IntraBio. A.C., E.K., E.A., L.D., S.Z., C.Y.T., M.E.F.‐S., P.D., F.D.P. and B.B. declare that they have no conflict of interest.

## AUTHOR CONTRIBUTIONS

A.C. and E.K. devised and performed the majority of the experiments, with specific experiments performed or supported by E.A. and M.E.F.‐S. Acidic store calcium measurements were performed by L.D. and A.G. The Tangier disease cells were provided by C.Y.T., P.D., S.Z., A.D., and B.B., along with mutation analysis. NPC1 fibroblasts were provided by F.D.P. F.M.P. helped devise the experiments, interpret the data and write the paper with A.C. All authors reviewed and approved the final version of the manuscript.

## ETHICS APPROVAL AND CONSENT TO PARTICIPATE

Human ABCA1‐mutant fibroblasts, surplus to the requirements for diagnosis, were collected with informed consent during the diagnostic workup at the University Hospital‐Udine and Addenbrooke's Hospital, Cambridge. NPC1 patient cells were obtained from Dr Porter. All procedures followed were in accordance with the ethical standards of the responsible committee on human experimentation (institutional and national) and with the Helsinki Declaration of 1975, as revised in 2000 (5). Informed consent was obtained from all patients for being included in the study. Additional informed consent was obtained from all patients for which identifying information is included in this article.

## PATIENT CONSENT STATEMENT

Patients 1, 2, 4 all gave written informed consent for the publication of their details in this case report. Patient 3 was lost to follow up and so all potentially identifiable personal data has been redacted.

## DATA AVAILABILITY

Data sharing is not applicable to this article as no datasets were generated or analysed during the current study.
